# The Influence of Spinal Pain, Spinal Mobility, and Spinal Curvature on the Risk of Falling in Osteoporotic Patients

**DOI:** 10.3390/jcm14134511

**Published:** 2025-06-25

**Authors:** Antonia Diegisser, Janine Huthwelker, Jürgen Konradi, Friedrich Bodem, Philipp Drees, Ulrich Betz

**Affiliations:** 1 Institute of Physical Therapy, Prevention and Rehabilitation, University Medical Center of the Johannes Gutenberg University Mainz, Langenbeckstraße 1, 55131 Mainz, Germanyjuergen.konradi@unimedizin-mainz.de (J.K.); ulrich.betz@unimedizin-mainz.de (U.B.); 2 Department of Orthopaedics and Traumatology, University Medical Center of the Johannes Gutenberg University Mainz, Langenbeckstraße 1, 55131 Mainz, Germany; philipp.drees@unimedizin-mainz.de

**Keywords:** osteoporosis, fall hazard, spinal mobility, spinal kyphosis and lordosis, back pain

## Abstract

**Background/Objective:** Although the increased proneness to falling in osteoporotic patients has been stated in the literature, the knowledge of underlying reasons and their possible interactions is incomplete. For this reason, it was the aim of this work to investigate the possible relation between spinal pain, spinal mobility, and spinal curvature on the risk of falling in osteoporotic patients. **Methods:** Our study included a total of 100 osteoporotic patients. Standardized methods were used to evaluate spinal pain, spinal mobility, and spinal statics. The risk of falling was assessed by the Tinetti test. To explicitly determine potential spine-related risk factors for falling, the results observed were adjusted by linear regression statistics considering already known risk factors (e.g., age, level of activity, muscle weakness, disturbed lateral balance). **Results:** The risk of falling in osteoporotic patients was found to be influenced by spinal pain (*p* = 0.010), the total spine mobility (*p* = 0.013), and, in particular, by its rotational mobility (*p* = 0.019). Spinal curvature (spine inclination in this context) did not show a significant effect (*p* = 0.892). **Conclusions:** Spinal pain and total spine mobility, in particular its rotational mobility, contribute to the risk of falling in osteoporotic patients. This finding should be appropriately considered in preventive patient care programs.

## 1. Introduction

The risk of falling in the elderly population increases with age. It has been reported that almost one third of all people over 65 years fall at least once a year, while it is 50% in those above 80 years. With increasing age, the number of multiple falls per year increases [1,2,3]. It may be assumed that these numbers are actually much higher, as many falling incidents remain unreported. In many cases, a fall is kept secret by the person involved due to shame, fear, or trivialization. In 5% of all cases a fall results in fractures, in 10–15% it results in other severe injuries, and in up to 40% of cases it engenders admittance to a nursing home, and thus to dependence [4,5]. In Germany, it is estimated that the operative costs of fall-related fractures, the subsequent rehabilitation of those involved, and the fall-related care often needed for older persons exceed EUR 2 billion every year [6]. Currently, there are special groups for fall prevention measures. However, they focus almost entirely on the improvement of muscle strength and balance [7].

A challenge faced by a scientific approach to the falling problem results from the multiple underlying causes. The literature recognizes a range of already proven risk factors for falling. Among these are muscle weakness in the lower extremities, multi-medication, visual impairment, as well as disturbances in mobility or cognition [5,7,8]. These all represent typical symptoms of aging. However, the manner of how these individual impairments contribute to a combined effect or depend on each other is still widely unclear. The situation becomes especially critical with the presence of a similarly age-related osteoporosis. Worsening in bone stability is associated with an increased risk of injury caused by a fall. Furthermore, a number of studies have shown that patients with osteoporosis are more likely to fall than those unaffected by this condition [7,9,10]. However, the literature does not satisfactorily explain the causes of this observation. For this reason, the starting point for our study was the assumption that the possible reasons for an increased risk of falling are connected to the typical main symptoms of osteoporosis (spinal pain, spinal immobility, and hyperkyphosis) [7].

In some studies, back pain, as a common symptom of osteoporosis, has a significant influence on balance and therefore on the risk of falling for osteoporosis patients [11,12,13]. Chronic back pain limits certain movements. The level of activity is reduced, resulting in a decrease in muscle strength. Movements are less coordinated and less differentiated, potentially interfering with an appropriate response to equilibrium disorders. The limited movement possibilities of a hypomobile spine reduce the variety of equilibrium reactions. By kyphosing the spine, the body’s center of gravity shifts ventrally, which can result in increased instability of the body. As a consequence, stance and gait security are seriously impaired, which is possibly associated with an increased risk of falling, because of the body’s center of gravity shifting close to the border of the supporting area of the feet. These described symptom chains could create a vicious cycle of fear of falling and gait insecurity, causing falls and fractures, creating new anxiety [7].

Therefore, the aim of our study was the evaluation of potential spine-related risk factors, in particular of the possible relation between spinal pain, spinal mobility, and spinal curvature on the risk of falling in osteoporotic patients.

## 2. Materials and Methods

### 2.1. Subject Group

A total of 100 patients in the age range from 55 to 87 years suffering from osteoporosis participated in our study (female 87%, male 13%, mean age 70.8 years). Osteoporosis was diagnosed according to the criteria proposed by the WHO [14] (t-score below −2.5 SD, i.e., bone mineral density less than 2.5 SD below the peak bone mass of a young, healthy adult. The mean bone mineral density (measured at the femoral neck) of all patients investigated amounted to −3.05 SD). Exclusion criteria were defined to be acute bone fracture and/or disorders in mobility and cognitive function. Those items were assessed by an interview but were not further confirmed using X-ray or other standardized assessments.

The subjects were recruited at the weekly osteoporosis consulting hours of the University Medical Centre of the Johannes Gutenberg University Mainz as well as by public advertising with flyers and in local newspapers. The study was approved by the Ethics Board of the State Chamber of Medicine in Mainz (Ethics Commission of the State Chamber of Medicine in Rhineland Palatinate, Votumnumber 837.207.11(7749), date: 14 July 2011). All participants gave written informed consent prior to participating in the study.

### 2.2. Consideration of Risk Factors Already Acknowledged in Previous Studies

An increased tendency to fall in old age is not caused by one single factor only, but rather by various sicknesses and age-related functional impairments.

In this context, we thus took into account the following positively known fall risk factors in order to explicitly distinguish them from the subject’s osteoporotic and spine curvature/mobility condition considered in our study. The criteria age, usage of walking aids, number of previous falls, and intake of medication (intake of > 4 medications per day, intake of medication causing falling), were established in specific patient interviews. The level of activity was assessed using the Physical Activity of the Elderly Questionnaire (PASE). This questionnaire evaluates sporting activities and other daily activities typical for this age group such as gardening or housework [15]. Visual acuity was assessed using a Rosenbaum card, which is a scaled down version of the Snellen chart. The muscular strength of the lower limb was established using the Chair-Rising Test [7]. The Timed Up-and-Go Test (TUG) assesses the gait stability by the time a person needs to rise from a chair, walk three meters away, turn around, walk back to the chair, and sit down again [16]. A disturbance of lateral balance was determined using the tandem stand test [7]. For the assessment of back pain, a visual analog scale (VAS) was used. With regard to spinal curvature at upright stance we measured the kyphosis of the thoracic spine, the lordosis of the lumbar spine, and its inclination. The inclination was defined here as the shift in the body’s center of gravity in the ventral direction. The mobility of the spinal column was analyzed as the extent of its thoracic and lumbar rotation, its lateral flexion, and its extension–flexion movement. Spinal curvature, inclination, and flexion–extension movement were measured at an upright relaxed stance posture using the SpinalMouse^®^ system (Idiag, Zürich, Switzerland) (named Idiag M360^®^ since March 2018) with a computer-assisted and non-invasive device applied to the body surface. This device provides objective data about the angular orientation of each of the thoracic and lumbar vertebrae in the sagittal plane [17]. The data set obtained can be used to additionally compute the inclination of the trunk and the extent of the movement between maximum flexion and extension [17]. Previous studies have shown that in measurements of spine curvature and motion in standing the SpinalMouse^®^ provides data correlating with respective radiographic measurements with r = 0.93 and r = 0.96, respectively [17,18].

Lateral flexion and rotation of the spinal column were measured using the plurimeter (also known as pluricompass). The pluricompass (invented and patented by Rippstein in 1970) is a combination of a liquid-damped inclinometer and a compass needle that is simultaneously used for rotational measurements in the transverse plane. In the literature, its successful use has been reported with a plurimeter helmet for the measuring of the flexibility of the cervical spine in all three degrees of freedom (rotation, lateral flexion, and extension–flexion). In this application, it has shown a good intra-rater reliability (r = 0.87–0.96) [19,20]. Each of our measurements is carried out in a standardized sitting posture with a motion control procedure involving an isolated movement and measurement of the rotation of the thoracic and lumbar spinal column without any simultaneous movement of the hip joint. Two consecutive experimental runs were carried out, the first one for the subject’s instruction, the second one for the actual measurement.

### 2.3. Determination of the Risk of Falling

The risk of falling was determined indirectly by the Tinetti test [3]. This test assesses static and dynamic balance as well as gait according to a number of qualitative aspects. Sixteen different criteria in a variety of activities (sitting, standing up, standing, walking, turning, and sitting down) are included in this analysis to yield an individual ability score. According to this test, a score of less than twenty indicates an increased risk of falling [3].

### 2.4. Statistical Analysis

Sample size was calculated using the program nQuery^©^ 9.4 (Boston, MA, USA). With a power of 80%, we obtained a sample size of *n* = 98. An adjusted coefficient of determination of 0.5 was assumed from the model of the known variables influencing the risk of falling. An increase in the coefficient of determination of 0.05 was taken as an increase in potential risk factors. In the statistical analysis, we focused on the following three questions:

1. Does back pain influence the risk of falling in osteoporotic patients?
2. Does spinal curvature (inclination) influence the risk of falling in osteoporotic patients?
3. Does spinal mobility (rotation, lateral flexion, extension, ventral/dorsal flexion) influence the risk of falling in osteoporotic patients?

**Figure 1:**
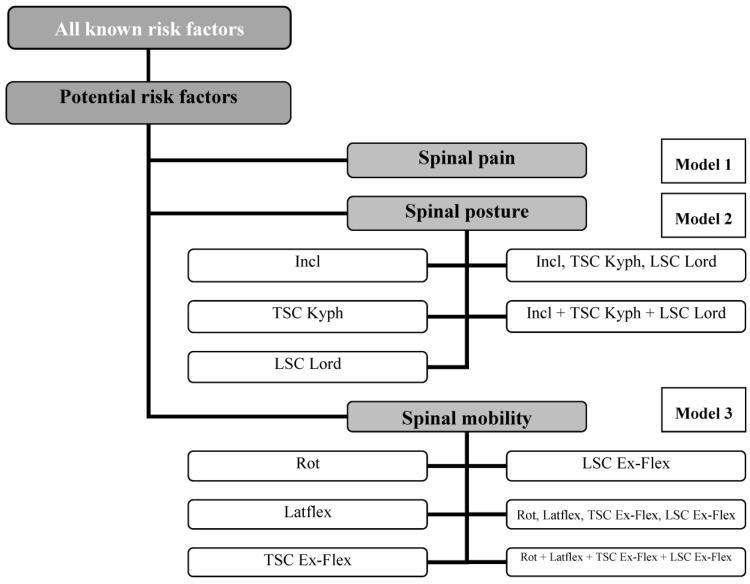
Distribution of the linear regressions of models 1–3. SC: Spinal column, Incl: Inclination, Kyph: Kyphosis, Lord: Lordosis, TSC: Thoracic spinal column, LSC: Lumbar spinal column, Rot: Rotation, Latflex: Lateral flexion, Ex-Flex: Extension–Flexion.

These are the questions being investigated in this study, so, the Bonferroni-corrected local level of significance must be set, in this case, at α = 0.05/3 = 0.017. Statistical analysis was performed using linear regression. In order to organize the data collected, we developed three regression models. They combined scientifically acknowledged risk factors with the potential spinal-related risk factors additionally considered in our study (model 1: back pain; models 2.1–2.5: spinal curvature; models 3.1–3.6: spinal mobility) (see Figure 1). In order to deal effectively with the multi-causality of the issue of falling, the data of the three potential risk factors—back pain, spinal curvature, and spinal mobility—were adjusted in the statistical analysis to the scientifically acknowledged risk factors. All data were analyzed using the Statistical Package for the Social Sciences (SPSS), (IBM, Armonk, NY, USA) Version 20.0 software.

## 3. Results

According to Table 1, 21 of the 100 individuals included in this study reported suffering at least one fall in the previous six months. A total of 40 subjects carried out moderate physical activities (level 3), most of them women, who were often members of gymnastics groups. They went shopping on foot or by bicycle or carried out light gardening or household work. Many more women reported pain in the spine or in the extremities compared to men. Overall, one third of all subjects reported light back pain (32%), but only 13% reported pain in the extremities, mostly in the knee. The median value for pain as assessed on the VAS was 3, representing “light pain”. A number of further subject-related findings are listed in detail in Table 1.

Table 2 shows the results of the motoric tests used to assess the risk of falling. About half of the subjects (53%) showed a loss of strength in the thigh as measured by the Chair-Rising Test. Women were more likely to exhibit muscle weakness than men (women 55.2%, men 38.5%, *p* = 0.283). Women were also more likely to exhibit a deficit in lateral balance, as measured by the tandem stand (women 39.1%, men 30.8%, *p* = 0.57). A fifth of the subjects suffered from both insecure gait and disturbance of dynamic or static balance (TUG 19%; Tinetti test 20%).

Table 3 shows the results of the static and dynamic spinal column measurements. The full amplitudes of the two directions of movement (left to right and front to back) are quantities chosen to represent the extent of the spatial mobility of the spine.

**Figure 2:**
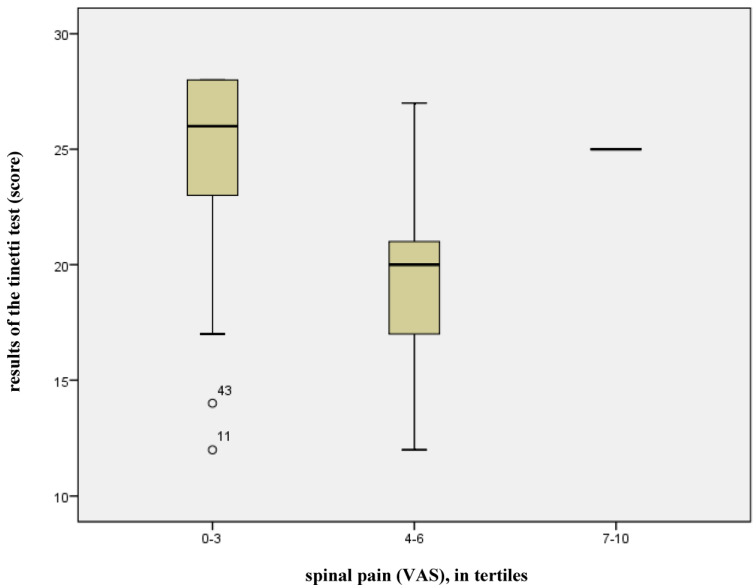
Effects of back pain on the results of the Tinetti test.

Back pain had a statistically significant influence (*p* = 0.010) on the risk of falling for patients with osteoporosis. The gradient of the respective regression line (−1.552) illustrates the clinical relevance of this factor, implying that an increase on the pain scale by one point reduces the score of the Tinetti test by 1.5 points. A total of 68% of the variance in falling risk can be attributed to model 1, i.e., to already scientifically acknowledged risk factors + back pain (adjusted R^2^ = 0.678, see Figure 1). The remaining 32% can be attributed to other factors which were not taken into account in this model. The box-plot diagram (see Figure 2) shows the relationship between back pain and the results of the Tinetti test, illustrating the effect of a low level or absence of back pain (VAS 0–3, *n* = 85) on the Tinetti test. Point scores of more than 20 indicate good balance and secure gait, while scores of 20 or less indicate a risk of falling. In the diagram, the scores for back pain, as expressed on the VAS, were divided into tertiles. The high point score of 7 yielded by the Tinetti test on the VAS can be considered an exception, as it relates to one single subject only.

In this evaluation, the spinal curvature is represented by its inclination. No evidence could be found that this factor has a statistically significant influence on the risk of a fall (*p* = 0.892), in addition to the known risk factors.

**Figure 3:**
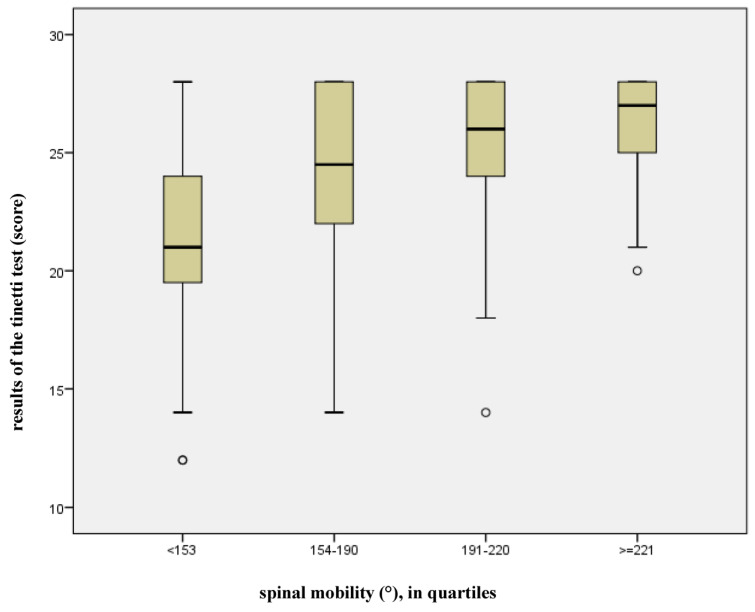
Effect of spinal mobility on the results of the Tinetti test.

Spinal mobility, expressed as the sum score of all independent motion amplitudes of the spine (rotation, lateral flexion, sagittal flexion/extension) showed a statistically significant influence on the risk of falling (*p* = 0.013). Furthermore, the examination of the linear regression line revealed that 64% of the variance in the risk of falling can be attributed to model 3 (previously scientifically acknowledged risk factors and spinal mobility, adjusted R^2^ = 0.640, see Figure 1). The remaining 36% can be attributed to other factors which were not taken into account in this model. Figure 3 shows a box-plot diagram of the relationship between overall mobility and the risk of falling. For this purpose, the total spinal mobility was divided into quartiles, with each assigned to the Tinetti test point score. It is evident that balance ability and security of gait increase in the Tinetti test point score (maximal value 28, cut-off-value ≤ 20 points) in proportion to the mobility of the spinal column.

**Figure 4:**
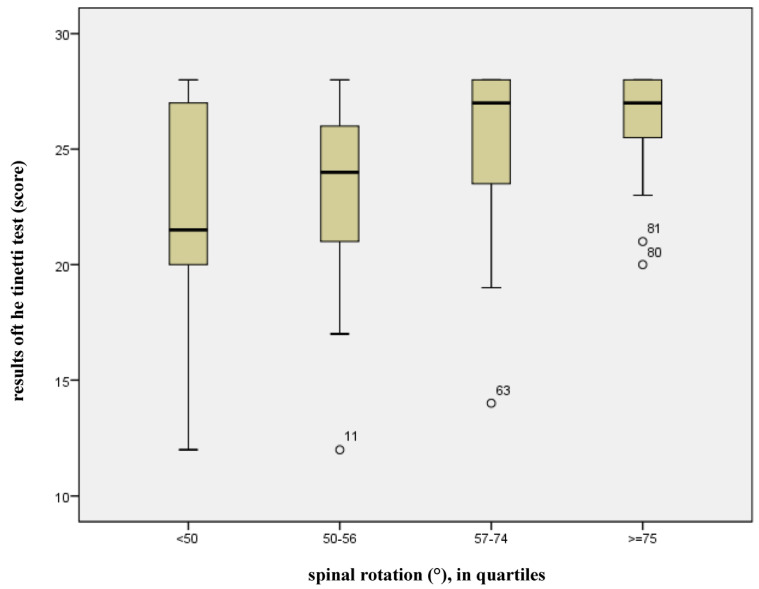
Effect of spinal column rotation on the results of the Tinetti test.

If the spinal mobility modes are considered separately, it is apparent that, in addition to the already acknowledged risk factors, only spinal rotation has a significant effect on the risk of falling (*p* = 0.019). Figure 4 illustrates how the point score of the Tinetti test increases in proportion to spinal rotation. The larger the rotation amplitude, the better the balance and security of gait. The risk of falling arises if the cut-off value of 20 points is not achieved in the Tinetti test. The widest spread of spinal rotation occurs in the lowest quartile (<50°, *n* = 24), but few subjects fall below the cut-off-value (median = 22 points). Only 25% of the subjects in this quartile achieved a point score ≤ 20 (smallest value 12).

## 4. Discussion

The present study shows that both back pain and spinal mobility, particularly the rotation amplitude, have a statistically significant influence on the risk of falling for patients with osteoporosis. No such influence could be shown for spinal inclination.

Back pain is a common symptom of osteoporosis [11,12,13,21]. Although at the time our study was carried out, only 32% of the included osteoporosis patients reported back pain, our statistical analysis revealed that back pain had a significant influence on balance and, therefore, on the risk of falling for these patients. This is in agreement with the findings of numerous studies published in the past [11,12,13]. In comparison to the other spinal parameters (mobility and curvature) examined in this study, statistically, back pain had the largest influence on the risk of falling. In the literature, it has often been reported that kyphotic spinal inclination is responsible for back pain in osteoporosis patients [11,13,22,23]. In fact, 66% of the patients complaining of back pain at the time of our study exhibited a progressed kyphosis. In the 65% of the patients in our study suffering from back pain, an inclination larger than 0° was seen, and in half of them it was larger than 5°. Studies examining the risk of falling in osteoporotic patients have also reported a connection between pain and a restriction of spinal column mobility. In all of these studies, however, only the extension–flexion movement was examined [23,24,25]. The subjects in our study complaining of pain at the time of examination also showed an average sagittal lumbar spinal column mobility amplitude of 37.9°, which was significantly less than in those not reporting pain (mean 46.5°, *p* = 0.006). We also observed a significant difference in spinal rotation between patients with back pain and those without it at the time of our study (spinal rotation of patients with back pain: 50°, spinal rotation of patients without back pain: 61.9°, *p* = 0.004). The consequences of chronic back pain are reduced physical activity as well as muscle weakness [11,13]. Since osteoporotic patients in the present study were only asked about their current pain and not about its duration, no statement can be made about the degree of pain chronicity. Of the 32 subjects complaining of back pain at the time of examination, almost half (46%) were assigned to activity levels 1 and 2, representing “very little” or “little” physical activity. Moreover, 75% of these subjects showed weakness in the thigh muscles during the Chair-Rising Test. The subjects were asked if they had pain outside the spine during the measurement. However, they were not explicitly asked about knee or foot pain, which can certainly have a considerable influence on gait and stance stability, and, consequently, on the risk of falling.

Spinal mobility, expressed as the sum score of the independent motion amplitudes (rotation, lateral flexion, extension/flexion), had a statistically significant effect on the risk of falling in osteoporotic patients. Considering the spinal mobilities separately, only the spinal rotation amplitude showed a statistically significant influence. Lateral flexion has no apparent effect. The extent of rotational mobility of the spinal column might be of great importance for maintaining balance. A study performed by American physiotherapists reported that a significant reduction in spinal rotation was observed in subjects who had fallen one or more times within a year. They established that the risk of falling increased 2.2 times if rotational mobility was reduced by 30° [26]. Axial rigidity or a limitation in axial rotation increases lateral instability, which may result in a great fall risk. This was also revealed by the study of Yang et al. [27] with adults suffering from Parkinson’s disease. The participants performed the Timed Up-and-Go Test and were recorded by a 3D motion capture system. Axial rotation was evaluated by the rotation onset of the head, thorax, and pelvis [27]. Former studies investigating spinal mobility in osteoporotic patients only evaluated the influence of the extension/flexion amplitude, not the effects of rotation and lateral flexion mobility deficits on the risk of falling. This may be due to the lack of practicable measuring devices. In our present study, we used the plurimeter and pluricompass, as introduced by Rippstein, for the measurement of the rotation and lateral flexion amplitudes. These manually applied devices have excellent reliability and even showed high correlation with the X-ray image [19,20]. However, a more precise digital measurement of lateral flexion would have been possible with the more modern version of SpinalMouse^®^ (IdiagM360) or with apparatus-based spinal analysis devices, but with a considerable additional cost in terms of acquisition and practicality.

The current study focused on the relationship between spinal column mobility and curvature parameters and the risk of falling in osteoporotic patients. In further studies, it would be interesting to investigate how the risk of falling is influenced by mobility in the lower extremities (hips, knees, and foot joints) and by deformities of the feet typical of this age group (hallux valgus, hammer toe, etc.). Some evidence of such an effect has been given in a study by Chiacchiero et al. [28] They showed that the respective given ranges of motion, in particular, the dorsal extension of the foot and of the hip abduction, and inner rotation and extension, were significantly less in a group that had suffered falls (≥2 falls in the last 12 months) compared to a group without fall incidents [28].

A kyphosis with inclined upper body posture as a risk factor of falling could not be confirmed in our study. The reason could be that the subjects included in this study showed an average spinal inclination only of 2° (range—7° and 17°), thus being without significant clinical importance. Looking more closely at the distribution of our data, it is apparent that 50% of the subjects exhibited a ventrally inclined posture, 36% of the subjects exhibited a dorsal tilt, and only 14% showed 0° of inclination. In studies reporting a positive correlation between inclination and balance, osteoporotic patients showed an average inclination of 6.81° [22]. In healthy older subjects, an average forward tilt of 17.1° was found [29]. The reason for the low inclination observed in the patient group of our study may be explained by the fact that they were still physically very active. An examination with the questionnaire PASE placed almost 2/3 (69%) of them in the two highest (moderate or high physical activity) activity levels. Some of them traveled a long distance in order to participate in the study, with almost all of them traveling independently by public transport or in their own cars. Activities of this type provide hints of a certain general degree of physical agility.

Because of this very physically active study population, the presented findings have to be interpreted with caution, as they might not be fully generalizable to all osteoporotic patients.

Furthermore, it has to be considered that the chosen study design only allows for associations between spinal pain and mobility and the risk of falling. Future research is required to investigate whether impaired spinal mobility and spinal pain are also causative factors for future falls in osteoporotic patients.

An important further aspect to consider is that the fall of an osteoporotic patient is nearly always a multi-causal incident. Both the literature and practical clinical experience provide a series of already demonstrated risk factors for falling, such as deficits in the muscle performance in the lower limbs or in lateral balance [5,7,8]. Other pathologies and impairments that have not been explicitly defined as exclusion criteria for participation might, therefore, also have influenced the risk of falling in the current study (e.g., neuropathy, osteoarthritis, chronic back pain, etc.). 

## 5. Conclusions

Through its more comprehensive approach to the problem area associated with the risk of falling, our study has been able to demonstrate that, in osteoporotic patients, the influence of back pain and impaired spinal mobility, in particular, impaired spinal rotation, has to be considered in the design of fall preventing programs. As possible additions to the usual contents of fall prevention programs, relaxation methods against back pain should be added, for example, breathing techniques or progressive muscle relaxation, as prescribed by Jacobson. In addition, exercises for increasing the mobility of the spine, especially those for rotation, must be undertaken. Among specifically gymnastics exercises, there are also playful measures for group treatments with balls, parachutes, etc. Further studies should also consider the reaction speed of the patient’s spinal column and the upper and lower extremities, as an impending fall requires a fast and effective response as a protective mechanism, either to avoid it altogether or to attenuate the impact, and thus avoid possible fractures.

## Figures and Tables

**Table 1:** Overview of the independent risk factors for falling.

		Women *n* = 87	Men *n* = 13	Total *n* = 100
**Number of falls in the last 6 months**	0 fall	62 (71.3%)	8 (61.5%)	70
	1 fall	18 (20.7%)	3 (23.1%)	21
	2 falls	5 (5.7%)	1 (7.7%)	6
	4 falls	1 (1.1%)	1 (7.7%)	2
	6 falls	1 (1.1%)	0	1
**Use of walking aids**		10 (11.5%)	1 (7.7%)	11
**Reduction in visus acuity**		33 (37.9%)	2 (15.4%)	35
**Intake of medications (>4 per day)**		26 (29.9%)	6 (46.2%)	32
**Fall-related drugs**		16 (18.4%)	1 (7.7%)	17
Level of activity *	level 1	11 (12.6%)	0	11
	level 2	16 (18.4%)	4 (30.8%)	20
	level 3	36 (41.4%)	4 (30.8%)	40
	level 4	24 (27.6%)	5 (38.5%)	29
**Spinal pain at the time of examination?**		30 (34.5%)	2 (15.4%)	32
**Pain in the extremities at the time of examination?**		12 (13.8%)	1 (7.7%)	13
**Dizziness at the time of examination?**		0	0	0
**Other influencing factors at the time of examination?**		0	0	0

* level 1: very little physical activity, level 2: little physical activity, level 3: moderate physical activity, level 4: much physical activity.

**Table 2:** Results of the motoric tests used to assess the risk of falling.

	MV	SD	Range	Test Positiv	MV	SD	Range	Test Positiv
	All, *n* = 100				Women, *n* = 87			
**Chair-Rising Test (seconds)**	12.2	5.9	0–40	53% ^1^	12.2 *	5.6	0–40	55.2%
**Tandem Test (seconds)**	11.0	5.4	0–15	38% ^2^	11.1 *	5.3	0–15	39.1%
**Timed Up-and-Go Test (seconds)**	9.8	4.2	5–29	19% ^3^	9.8 *	4.1	5–29	19.5%
**Tinetti Test** **(score)**	24	4	12–28	4% ^4^	24 *	4	12–28	19.5%

MV: mean value, SD: Standard deviation. ^1^ cut-off-value ≥ 11 s, ^2^ cut-off-value ≥ 10 s, ^3^ cut-off-value ≥ 12 s, ^4^ cut-off-value ≤ 20 Points. * Gender difference *p* > 0.05.

**Table 3:** Results of the measurement of spinal column mobility and static curvature.

	MV	SD	Range	MV	SD	Range	MV	SD	Range
	Women, *n* = 87			Men, *n* = 13			All, *n* = 100		
**SC lateral flexion (degree)**	70	21	20–110	75 *	28	15–122	70	22	15–122
**SC rotation** **(degree)**	57	19	20–115	62 *	17	20–80	58	19	20–115
**TSC** **extension–flexion (degree)**	14	15	−19–43	16 *	14	−2–53	14	14	−19–53
**LSC** **extension–flexion (degree)**	44	18	−12–78	41 *	18	13–78	44	18	−12–78

MV: mean value, SD: standard deviation, SC: Spinal column, TSC: thoracic spinal column, LSC: lumbar spinal column, * gender difference *p* < 0.05.

## Data Availability

The datasets presented in this article are not available because of legal restrictions.
